# Metallothionein 3 Potentiates Pulmonary Artery Smooth Muscle Cell Proliferation by Promoting Zinc-MTF1-ATG5 Axis-mediated Autophagosome Formation

**DOI:** 10.7150/ijbs.92992

**Published:** 2024-05-11

**Authors:** Tianxin Xiong, Yi Li, Molin Yang, Bo Huo, Xian Guo, Liyuan Liu, Yanxin Huang, Xuehai Zhu, Qinghua Hu, Xiang Wei, Ding-Sheng Jiang, Xin Yi

**Affiliations:** 1 Division of Cardiovascular Surgery, Tongji Hospital, Tongji Medical College, Huazhong University of Science and Technology, Wuhan, Hubei, China;; 2 Department of Cardiology, Renmin Hospital of Wuhan University, Wuhan, China.; 3 Key Laboratory of Organ Transplantation, Ministry of Education; NHC Key Laboratory of Organ Transplantation; Key Laboratory of Organ Transplantation, Chinese Academy of Medical Sciences, Wuhan, Hubei, China.; 4 Key Laboratory of Pulmonary Diseases of Ministry of Health of China, Wuhan, China.; 5 Department of Pathophysiology, School of Basic Medicine, Huazhong University of Science and Technology, Wuhan, China.

**Keywords:** Pulmonary artery smooth muscle cells, Metallothionein 3, Zn^2+^, MTF1, ATG5, Autophagosome formation

## Abstract

Abnormal proliferation of pulmonary artery smooth muscle cells (PASMCs) is one of the critical pathological mechanisms of pulmonary hypertension (PH), and therefore is gradually being adopted as an important direction for the treatment of PH. Metallothioneins (MTs) have been reported to be associated with PH, but the underlying mechanisms are not fully understood. Here, we demonstrated that the expression level of metallothionein 3 (MT3) was significantly increased in pulmonary arterioles from PH patients and chronic hypoxia-induced rat and mouse PH models, as well as in hypoxia-treated human PASMCs. Knockdown of MT3 significantly inhibited the proliferation of human PASMCs by arresting the cell cycle in the G1 phase, while overexpression of MT3 had the opposite effect. Mechanistically, we found that MT3 increased the intracellular zinc (Zn^2+^) concentration to enhance the transcriptional activity of metal-regulated transcription factor 1 (MTF1), which promoted the expression of autophagy-related gene 5 (ATG5), facilitating autophagosome formation. More importantly, MT3-induced autophagy and proliferation of human PASMCs were largely prevented by knockdown of MTF1 and ATG5. Therefore, in this study, we identified MT3-Zinc-MTF1-ATG5 as a novel pathway that affects PASMC proliferation by regulating autophagosome formation, suggesting that MT3 may be a novel target for the treatment of PH.

## Introduction

Pulmonary hypertension (PH) is a life-threatening health problem for people worldwide, with current studies estimating the prevalence of PH at approximately 1% of the global population 1, and as high as 10% in people over the age of 65 2. The advanced prognosis of PH is very poor, mainly due to increased pulmonary vascular resistance caused by progressively elevated pulmonary artery pressure, which further leads to pulmonary vascular remodeling (PVR) and right ventricular hypertrophy (RVH), and ultimately to right heart failure 3, 4. Reversal of PVR is fundamental to improve the prognosis of PH, which is mainly characterized by progressive occlusion of pulmonary arteries due to abnormal proliferation of pulmonary artery smooth muscle cells (PASMCs) 5. Therefore, investigating the molecular mechanisms of abnormal PASMC proliferation during PH is likely to be key to the development of pharmacological treatments for PH.

Autophagy is an autocatabolic process that plays a role in protecting cells by degrading misfolded proteins and damaged organelles 6, 7. Interestingly, autophagy plays an important role in PH 8, but the exact effect of autophagy in PH is still controversial 9. Several studies have demonstrated that autophagy can be activated by hypoxia in PASMCs 8, 10, 11. Some researchers have also found that chloroquine inhibits the proliferation of PASMCs and prevents PVR and RVH as well as the development of PH by inhibiting the autophagy pathway and inhibiting the degradation of lysosomal BMPR2 protein 11. In contrast, other studies have shown that PH susceptibility is increased in LC3B knockout mice and that PASMC proliferation is inhibited after LC3B knockdown 12. In addition, mTOR, as one of the inhibitors of the autophagy pathway, has been reported to activate the abnormal proliferation of PASMCs and pulmonary artery endothelial cells (PAECs), possibly by inhibiting autophagy, and can be reversed by the mTOR inhibitor rapamycin 13-15. Since autophagy is a dynamic process, this seemingly paradoxical phenomenon may be related to the fact that different stages of autophagy are regulated during PH, and the specific mechanisms require further investigation.

Metallothionein 3 (MT3), a member of the metallothionein family, is mainly involved in the maintaining intracellular homeostasis of metal ions such as Zn and Cu 16. MT3 was first identified as a potential neuronal growth inhibitory factor that could promote neurological recovery in Alzheimer's disease patients by inhibiting neuronal growth 17, 18. Subsequently, some studies reported that MT3 could regulate the proliferation of tumor cells, but showed different effects in different tumor cells 19-21. On the one hand MT3 could inhibit the proliferation of acute myeloid leukemia cells by upregulating forkhead box O1 (FOXO1) 21, while on the other hand, MT3 overexpression promoted the proliferation and migration of bladder cancer cells and breast cancer cells 22, 23. Thus, these studies indicate that the role of MT3 in proliferation regulation is controversial. Furthermore, it is unclear whether MT3 is involved in the regulation of PASMC proliferation and the development of PH. In addition, it has been reported that Zn^2+^ can activate autophagy by increasing Beclin1 expression through the ERK1/2 signaling pathway or promote autophagy by mediating the entry of metal-regulated transcription factor 1 (MTF1) into the nucleus to activate the transcription of autophagy-related genes (ATGs) 24, 25. However, it is currently unknown whether MT3 can affect the proliferation of PASMCs by regulating autophagy via Zn^2+^.

In the present study, we demonstrated that the expression of MT3 was significantly increased in pulmonary arterioles from PH patients and hypoxia-induced rats or mice. MT3 knockdown inhibited, while MT3 overexpression promoted, the proliferation of PASMCs by regulating zinc-MTF1-ATG5 pathway-mediated autophagosome formation. Our findings revealed a novel molecular mechanism regulating the proliferation of PASMCs that contributes to the development of PH, which may provide potential targets for the treatment of PH.

## Methods and materials

### Human samples

All experiments involving human specimens were conducted after approval by the Human Research Ethics Committee of Tongji Hospital, Tongji Medical College, Huazhong University of Science and Technology, and informed consent was obtained from the patients. Lung tissues in the PH group were obtained from patients with PH due to lung disease and/or hypoxia who underwent lung transplantation, and lung tissues from donors without lung disease for the control groups. Small pulmonary arteries (outer diameter <1 mm) were isolated from PH patients and donors, and all specimens were immersed in 4% paraformaldehyde or stored in a -80°C refrigerator prior to subsequent experiments.

### Animal models of PH

The method of constructing the chronic hypoxia models in rats and mice was essentially the same as our previous studies 26, 27. All animal experiments in this study were approved by the Animal Care and Use Committee of Tongji Hospital, Tongji Medical College, Huazhong University of Science and Technology. Briefly, to establish a murine model of chronic hypoxia-induced PH, male Sprague-Dawley rats (180-200 g) and male C57BL/6 mice (20-25 g) were exposed to a mixture of 10% O_2_, 1% CO_2_, and 89% N_2_ air for 4 weeks (control groups exposed to normal atmosphere). In the monocrotaline (MCT)-induced PH model, male Sprague-Dawley rats (180-200 g) were given a single subcutaneous injection of 60 mg/kg MCT and then maintained under normoxic conditions for 3 weeks to induce PH 26. In the Sugen5416 combined with hypoxia (SuHx)-induced PH model, male Sprague-Dawley rats (180-200 g) were injected subcutaneously with 20 mg/mL Sugen5416 (HY-10374, MCE), maintained in hypoxia (10% O_2_ concentration) for 3 weeks and finally transferred to normoxia for additional 2 weeks 27. Hemodynamic measurement was performed at the end of modeling, and the data showed increased right ventricular systolic pressure 26. Rat and mouse lung tissues were then collected and immersed in paraformaldehyde for fixation, followed by dehydration and paraffin embedding. HE staining showed typical features of pulmonary artery remodeling during PH in both hypoxia-induced rats and mice 26.

### Plasmid construction

The MT3 overexpression plasmid (MT3-Flag) was synthesized by Beijing Tsingke Biotech Co., Ltd, and the MTF1 overexpression plasmid (MTF1-ORF, CH835264) was purchased from Shandong Weizhen Biotechnology Co., Ltd, which were then cloned into the pHAGE-Flag plasmid. Short hairpin RNAs (shRNAs) targeting human MT3, MTF1 and ATG5 were inserted into the PLKO.1 vector between the AgeI and EcoRI restriction enzyme sites. The target sequences are shown below: human-shMT3-1: 5'-CGCGGACTCCTGCAAGTGCGA-3'; human-shMT3-2: 5'-AGGGATGCAAATGCACCTCCT-3'; human-shMTF1: 5'-GCAGCAAGCCACCACCTTAAA-3'; human-shATG5: 5'-CTTTGATAATGAACAGTGAGA-3'.

### Cell culturing and treatment

Human pulmonary artery smooth muscle cells (HPASMCs) were purchased from Lonza Bioscience (Catalog#: CC-2581). The HPASMCs used in our experiments were cultured in DME/F12 medium (SH30023.01, HyClone) supplemented with 10% fetal bovine serum (SH30084.03, HyClone) and 1% penicillin-streptomycin (15140122; Thermo Fisher Scientific) and passaged every 2 days. HPASMCs were exposed to hypoxia to stimulate PH in vitro, as our previously reported 26. HPASMCs transfected with the indicated lentivirus were placed in a three-gas incubator with 1% O_2_, 5% CO_2_ and 94% N_2_ (control for 21% O_2_, 5% CO_2_, and 74% N_2_) for the indicated times. The HPASMCs were treated with ZnSO_4_ (50 μM, Z0251, Sigma-Aldrich) to investigate the role of zinc on proliferation.

### Lentivirus packaging and transfection

Lentivirus preparation and infection were performed as described in our previous studies 28, 29. Briefly, the target plasmids and the lentivirus packaging plasmids (pMD2.G (12259, Addgene) and psPAX2 (12260, Addgene)) were cotransfected together into HEK293T cells using liposomes and polyethyleneimine (764604, Sigma-Aldrich). After 24 and 48 hours, lentiviruses containing the target genes in the supernatant of the culture medium were collected and co-incubated with HPASMCs, thus introducing the target genes into HPASMCs. After 24 hours of lentiviral infection, the HPASMCs were starved for 8 hours and then subjected to the specific treatments for the experiments.

### CCK-8 assay

A cell counting Kit-8 (CCK-8, BS350A, Biosharp) assay was used to evaluate the proliferation capacity of HPASMCs. HPASMCs transfected with the indicated lentiviruses were seeded in 96-well plates at 3×10^3^ cells per well, and the cells were treated with normoxia/hypoxia for 48 hours. Then, 10 μL of CCK-8 reagent was added to 100 μL of DME/F12 in each well, and the 96-well plates were incubated at 37°C for 2 hours. Finally, the absorbance at 450 nm was measured using a BioTex Synergy HT microplate reader to reflect the cell proliferation capacity.

### EdU incorporation assay

The Cell-Light^TM^ EdU Apollo 567 In Vitro Kit (C10310-1, RiboBio) was used to perform the EdU incorporation assay as previously reported 26, 30, 31. Briefly, lentivirus-infected and starved HPASMCs were plated in 24-well plates at 1.5×10^4^ cells per well and incubated in normoxia/hypoxia incubators for 48 hours. EdU (50 μM) medium was then prepared from EdU stock solution at a ratio of 1:1000 and added to 24-well plates at 300 µL per well and co-incubated at 37°C for 2 hours. The HPASMCs were then washed with phosphate-buffered saline (PBS) and immediately fixed with 4% paraformaldehyde. Glycine (2 mg/mL) was used to neutralize the aldehyde, and after washing in PBS, the cell membrane was permeabilized with 0.5% Triton X-100. HPASMCs were then stained with 1× Apollo staining solution for 30 minutes to visualize replicating DNA, and 1× 4',6-diamidino-2-phenylindole (DAPI) was used to label cell nuclei. Cell fluorescence images were observed using an Olympus fluorescence microscope (BX53, Tokyo, Japan).

### LDH assay

The cytotoxicity LDH Assay Kit (CK12, Dojindo) was used to detect cell injury/death as described in our previous study 32. HPASMCs were infected with the indicated lentivirus for 24 hours, starved for 8 hours and then seeded in 96-well plates at 8000 cells per well. After 24 hours of normoxic/hypoxic treatment, 100 µL of working solution was added to each well and incubated for 20 minutes in the dark. Then 50 µL of stop solution was added to each well to stop the reaction and the absorbance of each well was immediately recorded at a wavelength of 490 nm using a multimode microplate reader. Finally, the LDH release from each well was calculated from the blank and the high control.

### Immunofluorescence staining

We followed the standard procedure for immunofluorescent staining of tissues and cells as reported in our previous studies 32-34. The primary antibodies used in this study include the anti-metallothionein 3 (anti-MT3, A20541, ABclonal, 1:100 dilution), anti-α-smooth muscle actin (anti-α-SMA, AB7817, Abcam, 1:200 dilution) and anti-vWF (66682-1-lg, Proteintech, 1:200 dilution). The expression level of MT3 in pulmonary arteries was presented by quantifying the mean fluorescence intensity (MFI) of MT3 positivity in α-SMA- or vWF-positive cells using ImageJ software. The α-SMA-labeled vessels smaller than 100 μm with an intact lumen are pulmonary arterioles. The thickness of the pulmonary artery tunica media was calculated as the result of (outer diameter-inner diameter)/outer diameter of the pulmonary artery tunica media under α-SMA labeling.

### Autophagic flux assay

GFP-mCherry-LC3 plasmid was introduced into HPASMCs for monitoring autophagic flux under the indicated treatments as previously described 30, 31. To further assess the effect of MT3 on autophagic flux, we overexpressed or knocked down MT3 in HPASMCs overexpressing GFP-mCherry-LC3, while 3-MA (5 μM, S2767, Selleck), a type III phosphatidylinositol 3-kinase (PI-3K) inhibitor, was used to inhibit autophagosome formation, and CQ (10 μM; C6628; Sigma-Aldrich), a lysosomotropic agent, was used to suppress autophagosome-lysosome fusion. After administration of the indicated stimulus, HPASMCs were fixed with 4% paraformaldehyde for 20 minutes and washed with PBS. Autophagic flux was then observed by a fluorescence microscopy. Yellow dots (GFP^+^/mCherry^+^) and red dots (GFP^-^/mCherry^+^) indicate autophagosomes and autolysosomes, respectively.

### Zn^2+^ Staining

To determine the effect of MT3 on Zn^2+^ levels, we examined Zn^2+^ levels in HPASMCs overexpressing or knocking down MT3 using Zinpyr-1. Lentivirus-infected and starved HPASMCs were first counted and plated in 24-well plates, followed by 48 hours of normoxic/hypoxic stimulation. At the end of stimulation, the HPASMCs were washed three times with PBS, then Zinpyr-1 solution (20 μM, Z276239, Aladdin) prepared in PBS was added to the well plates and incubated in the dark at 37°C for 30 minutes, after which the HPASMCs were washed again with PBS, and finally Zn^2+^ fluorescence images were immediately observed and collected under a fluorescence microscope.

### Flow cytometry

Flow cytometry assays were used for cell cycle, Zn^2+^ detection and ROS detection. For the cell cycle assay, at the end of the indicated stimuli, cells were collected by digestion with trypsin, washed with PBS, centrifuged for 10 minutes, resuspended in cold PBS, and fixed overnight at 4°C by adding pre-cooled 70% ethanol. The next day, the cells were washed twice with PBS and then stained with PI (P4864, Sigma-Aldrich) solution with ribonuclease A (R5125, Sigma-Aldrich) for 4 hours in the dark. Finally, stained cells were sorted on a BD FACSAria^TM^Ⅲ sorter and cell cycle analysis was performed using ModFit software.

For Zn^2+^ detection experiments, HPASMCs were infected with the indicated lentivirus for 24 hours, starved for 8 hours, and then subjected to normoxic/hypoxic stimulation for 48 hours. After washing with PBS, the cells were incubated with Zinpyr-1 solution (20 μM) at 37°C for 30 minutes. Residual solution was removed by washing the cells with PBS, followed by trypsinization, centrifugation, and washing again with cold PBS. The cells were then resuspended in 200 μL PBS. Finally, the mean fluorescence intensity of Zn^2+^ at 525 nm was detected in the BD FACSAria^TM^ III sorter, and FlowJo software (version 10.7.2) was used for flow cytometry analysis.

For ROS detection, HPASMCs infected with the indicated lentivirus were subjected to normoxic/hypoxic treatment for 48 hours. The diluted DCFH-DA probe (S0033S-1, Beyotime) was then incubated with these HPASMCs for 20 minutes. The HPASMCs were washed three times with serum-free medium and then harvested for sorting on the BD FACSAria^TM^ III sorter. Finally, analysis of fluorescence values under the FITC channel was performed using Flowjo software.

### Protein extraction and western blot

Western blot was performed as previously described 26, 29, 32. In brief, total protein was extracted from HPASMCs by using radioimmunoprecipitation assay (RIPA) lysis buffer [900 μL of RIPA, 10 μL of protease and phosphatase inhibitor cocktail (Thermo Fisher Scientific, 78440), 20 μL of PMSF, 10 μL of EDTA solution (AM9260G, Thermo Fisher Scientific), 50 μL of NaF, 10 μL of Na_3_VO_4_]. First, total protein was loaded and separated by 12% SDS-PAGE and then transferred to polyvinylidene fluoride (PVDF, IPVH00010, Millipore) membranes. The membranes were then blocked with 5% non-fat milk in a shaker for 1 hour at room temperature. After washed three times with tris buffered saline tween (TBST), the membranes were incubated with primary antibodies overnight at 4°C. Next day, the membranes were incubated with the corresponding species of peroxidase-conjugated secondary antibody for 2 hours at room temperature on a shaker after washed three times with TBST. Finally, protein signals were enhanced with chemiluminescent reagents and visualized in the ChemiDoc^TM^ XRS+ system (Bio-Rad). Antibodies used in this study included anti-β-actin (AC026, 1:50000, rabbit, ABclonal), anti-PCNA (GTX100539, 1:200, rabbit, Genetex), anti-p-Rb (#8516, 1:1000, rabbit, CST), anti-p21 (#2947, 1:1000, rabbit, CST), anti-LC3A/B (#12741, 1:1000, rabbit, CST), anti-p62 (ab56416, 1:300, mouse, Abcam), anti-ATG5 (10181-2-AP, 1:1000, rabbit, Proteintech), anti-ATG7 (67341-1-lg, 1:5000, mouse, Proteintech).

### RNA sequencing (RNA-seq) and analysis

HPASMCs infected with Lenti-PLKO.1 and Lenti-shMT3 and treated with hypoxia for 48 hours were harvested for RNA sequencing, which was performed by Novogene Co., Ltd. (Beijing, China) using the Illumina NovaSeq platform. After quantitative processing, the count files were imported into the R (version 4.1.2) package DEseq2 package and differentially expressed genes (DEGs) were analyzed based on a negative binomial distribution model. We used the Benjamini and Hochberg's method to adjust the *P*-value to improve accuracy. In addition, we screened for differentially expressed genes by an absolute value of log_2_(fold change) ≥ 0.7 and an adjusted *P*-value ≤ 0.05. According to DEGs, Gene Ontology (GO), Kyoto Encyclopedia of Genes and Genomes (KEGG), and Gene Set Enrichment Analysis (GSEA) were performed via the clusterProfiler (v3.16.1) R package.

### Real-time PCR

Real-time PCR (RT-PCR) was performed as previously described 30, 35. Total mRNA in HPASMCs or in small pulmonary arteries (outer diameter <1 mm) isolated from PH patients and donors was extracted using TRI Reagent^®^ Solution (A33251, Thermo Fisher Scientific) and then reverse transcribed into cDNA by using the Hifair^®^Ⅱ first stand cDNA Synthesis Kit (gDNA digester plus; 11119ES60, Yeasen). Target genes were amplified in CFX connect^TM^ real-time PCR detection system (Bio-Rad) by using iQ^TM^SYBR^®^ Green Supermix (1708884, Bio-Rad). The corresponding primers were as follows: 18S forward primer: 5'-CTCAACACGGGAAACCTCAC-3'; reverse: 5'-CGCTCCACCAACTAAGAACG-3'; MT3 forward primer: 5'-CACCTCCTGCAAGAAGAGCT-3'; reverse: 5'-GCACTTCTCTGCTTCTGCCT-3'; ATG5 forward primer: 5′-GCTTCGAGATGTGGTTTGG-3′; reverse: 5′-CCATTTCAGTGGTGCCTTC-3′.

### Luciferase reporter assay

The Luciferase reporter assay was performed as described in our previous studies 36, 37. The promoter sequence of the human ATG5 gene located between 106317821 and 106316221 was cloned into the pGL3-basic reporter vector. The pGL3-ATG5 luciferase reporter plasmid was constructed using primers 5'-TGCTAGCCCGGGCTCGAGTGCCAAATGCAGAAACTCAA-3' (forward) and 5'-TACCGGAATGCCAAGCTTCCCCCTGAACACCTTTCTCT-3' (reverse). For the luciferase assay, HEK293T cells were cotransfected with 0.2 μg of pENTER or MTF1-ORF, 0.04 μg of pGL3-ATG5 plasmid, and 0.02 μg of TK (Renilla luciferase) plasmid per well. After 24 hours of transfection, the cells were starved for 8 hours, and then cultured in high glucose medium with 10% FBS for 24 hours. Cells were lysed with 60 μL passive lysis buffer (PLB, E1910, Promega). Firefly and Renilla luciferase activities were then detected after treatment with Luciferase Assay Reagent II (LAR II) and Stop & Glo® fluorescence quencher, respectively, and the ratio of firefly (pGL3-ATG5) and Renilla (TK) activities represented the transcriptional activity.

### Statistical analysis

All data in this study were analyzed using GraphPad Prism 8 software. Continuous variables were expressed as mean ± standard deviation. Unpaired Student's t-test was used to indicate the difference between the means of the two groups, and multiple group comparisons were made using one-way ANOVA with Bonferroni and Tamhane's T2 post hoc test. *P*-value < 0.05 was considered as statistically significant difference.

## Results

### Metallothionein 3 (MT3) is Upregulated during PH and PASMCs Proliferation

**Figure 1:**
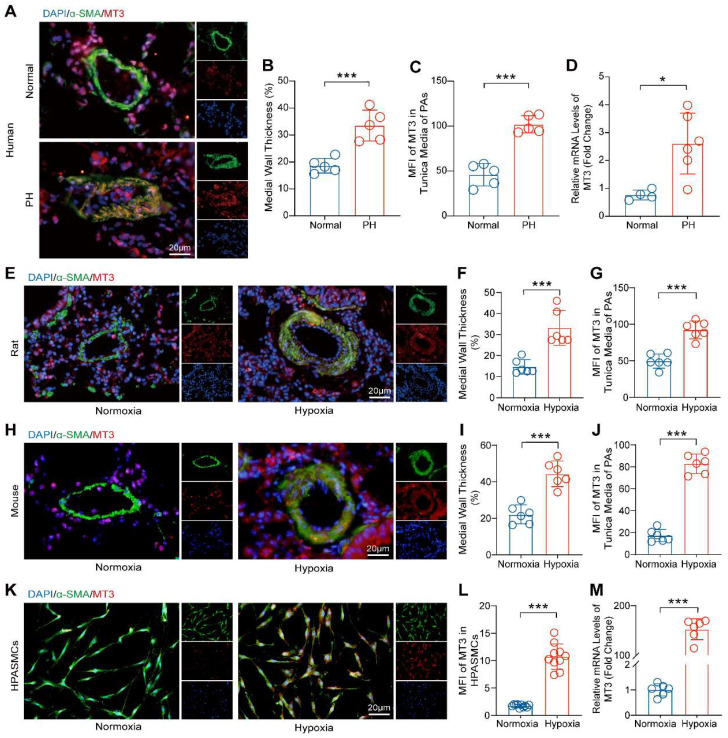
** MT3 expression is increased in the tunica media of pulmonary arteries from PH patients, chronic hypoxia-induced rat/mouse models, and hypoxia-treated HPASMCs. A**, Representative images showing immunofluorescence staining against α-SMA (green) and MT3 (red) in the pulmonary arteries (PAs) from normal and PH patients (scale bar, 20 μm). **B**, Quantification of medial wall thickness [(outer diameter-inner diameter)/outer diameter] of the PAs in the normal control and PH patient groups, α-SMA marks the tunica media of the pulmonary artery (n=5). **C**, Mean fluorescence intensity (MFI) of MT3 in the tunica media (quantified as the intensity of red fluorescence in the α-SMA-positive area) of PAs in normal controls and PH patients (n=5). **D**, MT3 mRNA levels in pulmonary arteries from normal and PH patients (normal control groups n=4, PH patient groups n=6).** E**, Representative images showing immunofluorescence staining against α-SMA and MT3 in PAs from normoxia and 4-week hypoxia-treated rats (scale bar, 20 μm). **F** and **G**, Medial wall thickness and MT3 expression levels in the tunica media of PAs were quantified in normoxia- and hypoxia-treated rats, and quantitative methods are consistent with **B** and **C** (n=6). **H**, Representative images showing immunofluorescence staining against α-SMA and MT3 in PAs of the mice treated with normoxia and 4-week of hypoxia (scale bar, 20 μm). The thickness of the tunica media and MT3 expression levels in PAs were quantified in **I** and **J** (n=6). **K**, Representative images showing cyto-fluorescence staining against α-SMA and MT3 from the HPASMCs treated with normoxia and 48 hours of hypoxia, and the expression level of MT3 was quantified in **L** (n=10, scale bar, 20 μm). **M**, MT3 mRNA levels in HPASMCs treated with normoxia and hypoxia 48 hours were detected by RT-PCR (n=6). *P<0.05, **P<0.01, ***P<0.001.

To investigate the involvement of MT3 in the process of PH, we first detected the expression pattern of MT3 in tissue samples from PH patients. The results of α-SMA and MT3 immunofluorescence showed that pulmonary arterioles of PH patients exhibited severe remodeling and muscularization compared with donors, and more importantly, the expression level of MT3 was significantly upregulated in the pathologically thickened tunica media of pulmonary arterioles of PH patients **(Figure 1A-1C)**. Moreover, RT-PCR results showed that MT3 mRNA levels in pulmonary arteries from PH patients were significantly higher than those in the normal group **(Figure 1D)**. To further validate this finding, the rat and mouse PH models were generated by chronic hypoxia (CH) treatment for 4 weeks 26. Similarly, it was shown that higher MT3 expression levels were detected in the tunica media of pulmonary arterioles in the CH treated group than in the normoxia group in both rats and mice **(Figure 1E-1J)**. In addition, given the important role of endothelial cells in PH and the fact that MT3 has been reported to be expressed in endothelial cells 4, 38, 39, we also examined whether MT3 expression was altered in endothelial cells during hypoxia-induced PH. The results showed that MT3 expression in the endothelium of pulmonary arterioles was comparable between normal and PH patients, as well as in normoxia- and hypoxia-treated rats **(Figure S1A-S1D)**. To further clarify whether the increased expression of MT3 in PASMCs during PH is etiology dependent, we constructed various etiology-induced PH rat models and examined the expression of MT3 in these models. The results showed that the expression of MT3 in the tunica media of pulmonary arterioles was significantly higher than that in normal controls in both the MCT-induced PH rat model and the SuHx-induced PH rat model **(Figure S2A-S2F)**, suggesting that MT3 may be involved in PH induced by different etiologies. Furthermore, human pulmonary artery smooth muscle cells (HPASMCs) were treated with hypoxia (1% oxygen concentration) for 48 hours to simulate the vascular microenvironment in PH patients *in vitro*. We found that an increased MT3 expression level was observed in hypoxia-treated HPASMCs compared with the normoxia group **(Figure 1K and 1L)**. In addition, hypoxia treatment also increased the MT3 mRNA level in HPASMCs **(Figure 1M)**. Thus, these results indicated that MT3 may be involved in the development of PH by regulating the function of PASMCs.

### Knockdown of MT3 Inhibits the Proliferation of PASMCs

**Figure 2:**
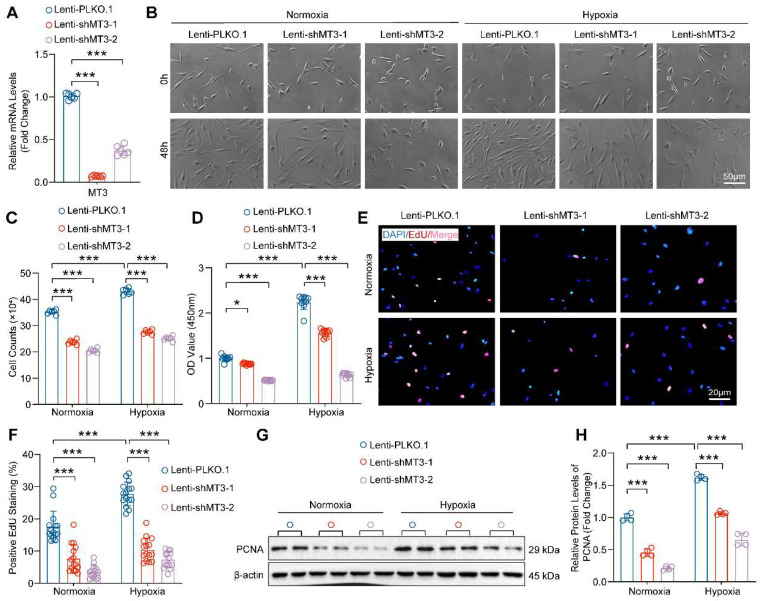
** MT3 knockdown suppresses HPASMC proliferation. A**, The mRNA levels of MT3 were detected by RT-PCR (n=6). HPASMCs were infected with Lenti-PLKO.1, Lenti-shMT3-1, and Lenti-shMT3-2 and cultured under normoxic or hypoxic conditions for the indicated times. **B**, Representative light microscopy images of HPASMCs were obtained at 0 and 48 hours (scale bar, 50 μm). **C**, The number of HPASMCs in control and MT3 knockdown groups was counted after 48 hours of culture (n=6). **D**, Cell viability of HPASMCs in the indicated groups was assessed by CCK-8 assay after 48 hours of culture (n=10). **E** and** F**, Representative images and quantitative analysis of EdU-positive HPASMCs from the indicated groups (n=15, scale bar, 20 μm). **G** and **H**, Western blot analysis and quantification showing the expression level of the proliferation marker PCNA (n=4). *P<0.05, **P<0.01, ***P<0.001.

To further investigate the role of increased MT3 in PASMC proliferation during PH, we constructed two different short hairpin RNA plasmids to knock down MT3 (Lenti-shMT3-1 and Lenti-shMT3-2) in HPASMCs, which resulted in significantly decreased MT3 mRNA levels in HPASMCs compared with Lenti-PLKO.1 **(Figure 2A)**. Compared with the Lenti-PLKO.1 group, infection with Lenti-shMT3-1 and Lenti-shMT3-2 significantly reduced the proliferation of HPASMCs under both normoxic and hypoxic conditions, as assessed by cell counting assay after 48 hours of culture **(Figure 2B and 2C)**. Furthermore, the results of both CCK-8 and EdU incorporation assays also showed that MT3 knockdown effectively inhibited the cell viability and proliferative capacity of PASMCs under both normoxic and hypoxic conditions **(Figure 2D-2F)**. In addition, the protein level of proliferation cell nuclear antigen (PCNA), a biomarker of cell proliferation, was significantly suppressed by MT3 deficiency in HPASMCs **(Figure 2G and 2H)**. Furthermore, LDH assay results showed that MT3 knockdown did not cause obvious HPASMC damage and death under normoxic or hypoxic conditions **(Figure S3A)**. Taken together, these results indicated that MT3 knockdown significantly inhibited the proliferation of PASMCs.

### Knockdown of MT3 Arrests PASMCs in G1 Phase

**Figure 3:**
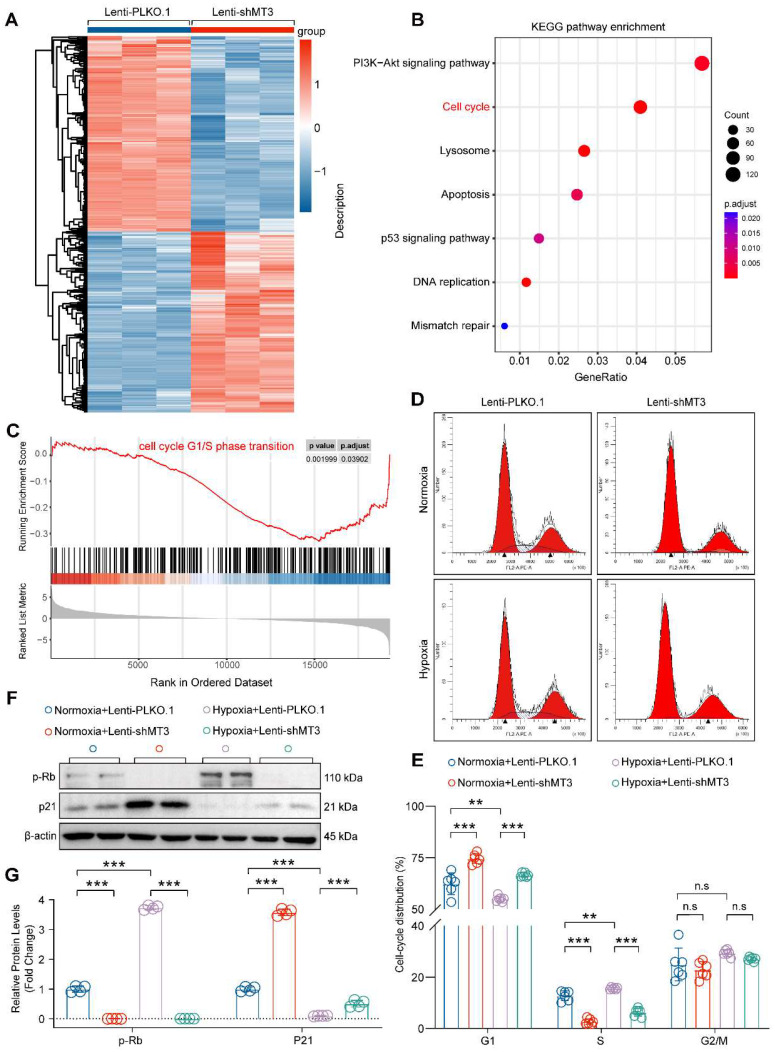
** MT3 knockdown inhibits the proliferation of HPASMCs by blocking the cell cycle in the G1 phase. A**, Heatmap showing the differentially expressed genes (DEGs) between control (Lenti-PLKO.1) and MT3 knockdown (Lenti-shMT3) HPASMCs (n=3). **B**, KEGG pathway enrichment analysis showing pathways and biological processes with these DEGs. **C**, Gene set enrichment analysis (GSEA) showing the reduction of G1/S phase transition. **D**, Representative images of flow cytometry of HPASMCs transfected with Lenti-PLKO.1 and Lenti-shMT3 subjected to normoxia/hypoxia treatment for 48 hours. **E**, Quantification of the ratio of HPASMCs in different cell cycle phases, including G1 phase, S phase, and G2/M phase (n=6). **F** and **G**, Western blot analysis and quantification showing the protein expression levels of G1-S checkpoint regulators including p-Rb and p21 in HPASMCs of the indicated groups (n=4). **P<0.01, ***P<0.001, n.s indicates no significant difference.

Next, transcriptomic sequencing was performed to dissect the biological processes regulated by MT3 knockdown to affect HPASMC proliferation. We first screened for differentially expressed genes (DEGs) using adjusted *P*-value ≤ 0.05 and log_2_(fold change) ≥ 0.7 criteria, which revealed a total of 5,467 DEGs, of which 2,628 were upregulated and 2,839 were downregulated after MT3 knockdown in HPASMCs **(Figure 3A)**. KEGG analysis revealed a significant enrichment of pathways related to cell proliferation, such as cell cycle and DNA replication **(Figure 3B)**. Furthermore, the result of GSEA showed that the gene set of cell cycle G1/S phase transition was enriched in HPASMC with MT3 knockdown **(Figure 3C)**. Based on these results, flow cytometry was performed after PI staining to determine whether MT3 knockdown affected the cell cycle of HPASMCs. The results showed that compared with the control group, MT3 knockdown resulted in more cells arrested in G1 phase and fewer cells entering S phase, suggesting that MT3 deficiency blocked HPASMCs at the G1/S cell cycle checkpoint **(Figure 3D and 3E)**. Since phosphorylated Rb (p-Rb) and p21 are the critical regulators of the G1-S transition during the cell cycle, their protein levels were determined by Western blotting. The results showed that the protein levels of p-Rb were downregulated, whereas the protein levels of p21 were upregulated in HPASMCs with MT3 knockdown** (Figure 3F and 3G)**. Taken together, these results demonstrated that MT3 knockdown inhibited the proliferation of HPASMCs by arresting them in G1 phase.

### Overexpression of MT3 Accelerates the Proliferation of PASMCs

**Figure 4:**
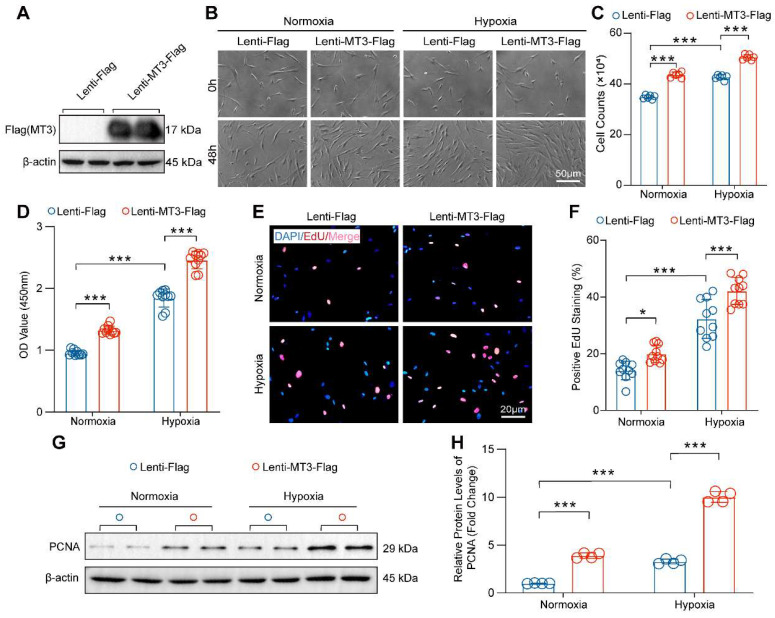
** MT3 overexpression promotes proliferation of HPASMCs. A**, MT3 expression levels were detected by Western blot in control (Lenti-Flag) and MT3-overexpressed (Lenti-MT3-Flag) HPASMCs. **B**, Representative light microscopy images of MT3-overexpressed HPASMCs cultured in normoxia/hypoxia for 0 and 48 hours (scale bar, 50 μm). **C**, The number of HPASMCs with or without MT3 overexpression was counted after normoxia/hypoxia treatment for 48 hours (n=6). **D**, CCK-8 assay was used to evaluate cell viability (n=10). **E** and **F**, EdU incorporation assay was performed to show the proliferation capacity of MT3-overexpressed (or not) HPASMCs (n=10, scale bar, 20 μm). **G** and **H**, Western blot analysis and quantification showing the protein level of the proliferation marker PCNA in HPASMCs of the indicated groups (n=4). *P<0.05, **P<0.01, ***P<0.001.

Then, MT3 was overexpressed in HPASMCs to further verify the effect of MT3 on proliferation **(Figure 4A)**. Compared with the control group (Lenti-Flag), overexpression of MT3 (Lenti-MT3-Flag) significantly promoted the proliferation of HPASMCs after 48 hours culture under both normoxic and hypoxic conditions **(Figure 4B and 4C)**. The result of CCK-8 assay also supported that MT3-overexpressing HPASMCs had higher cell viability than the control cells under normoxic and hypoxic conditions **(Figure 4D)**. Similarly, more EdU-positive cells were also detected in MT3-overexpressing HPASMCs than in control groups **(Figure 4E and 4F)**. In addition, higher protein levels of PCNA were detected in MT3-overexpressing HPASMCs compared to controls, further confirming the promoting effect of MT3 overexpression on PASMC proliferation **(Figure 4G and 4H)**. In contrast, the overexpression of MT3 did not lead to HPASMC injury and death under both normoxic and hypoxic conditions **(Figure S3B)**. Taken together, these results demonstrated that MT3 was a positive regulator of PASMC proliferation, which may be involved in pulmonary arterial remodeling and PH.

### Pro-proliferative Role of MT3 Largely Depends on Zn^2+^-activated MTF1 in PASMCs

**Figure 5:**
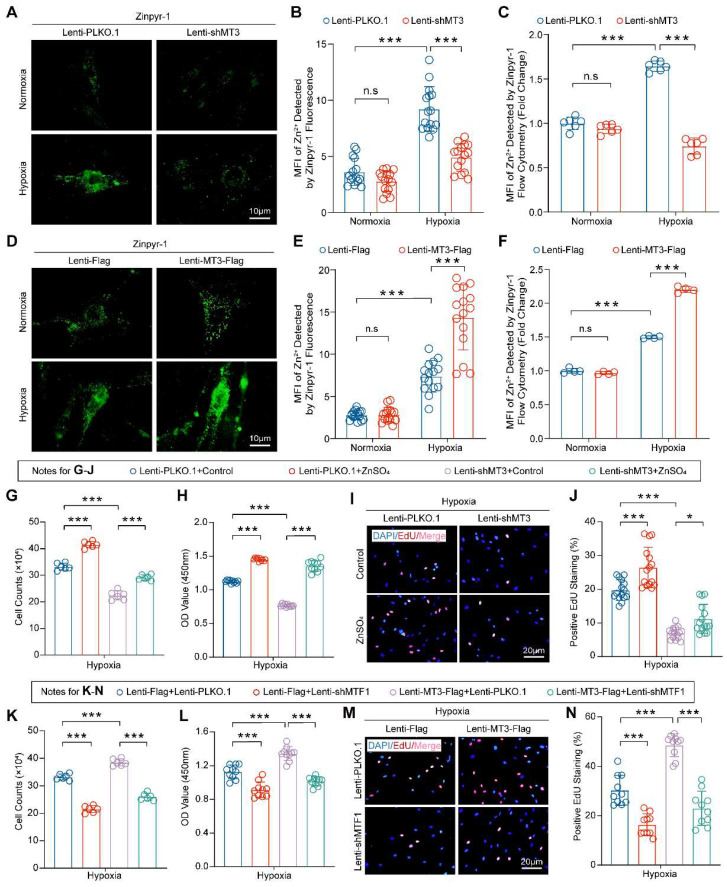
** MT3 promotes HPASMC proliferation by regulating the Zn^2+^-MTF1 axis. A and B**, Representative images showing Zn^2+^ fluorescence staining of MT3 knockdown HPASMCs subjected to 48 hours of normoxia/hypoxia treatment by a Zinpyr-1 probe and quantified in **B** (n=15, scale bar, 10 μm). **C**, Quantitative analysis of the mean fluorescence intensity (MFI) of Zn^2+^ in HPASMCs with the indicated treatments by flow cytometry (n=6). **D** and **E**, Representative images and quantitative analysis of Zn^2+^ fluorescence staining in MT3-overexpressed HPASMCs subjected to 48 hours of normoxia/hypoxia treatment by a Zinpyr-1 probe (n=15, scale bar, 10 μm). **F**, Representative images and quantitative analysis of the mean fluorescence intensity (MFI) of Zn^2+^ in HPASMCs with the indicated treatments by flow cytometry (n=4). Exogenous Zn^2+^ was supplied by 50 μM ZnSO_4_ co-incubation with HPASMCs MT3 knockdown or not. After all groups were treated with hypoxia for 48 hours, **G**, Cell counting assay was performed (n=6), **H**, CCK-8 assay was detected to assess cell viability (n=10), and EdU incorporation assay was performed to assess the proliferative capacity of the cells in **I** and **J** (n=15, scale bar, 20 μm). **K**, HPASMCs infected with Lenti-Flag and Lenti-MT3-Flag were subsequently reinfected with Lenti-PLKO.1 and Lenti-shMTF1, and number of cells counted after 48 hours of hypoxia. Under the same conditions as in **K**, CCK-8 (**L**) and EdU incorporation assays (**M** and **N**) were performed to determine cell viability and proliferation capacity, respectively (n=10 for CCK-8 and EdU, scale bar, 20 μm). *P<0.05, **P<0.01, ***P<0.001, n.s indicates no significant difference.

Numerous studies have suggested that MT3 is an important redox protein and that its cysteine sulfhydryl group releases hydrogen to bind oxygen radicals, thereby reducing reactive oxygen species (ROS) in cells such as osteoblast or adipocytes 40, 41, and ROS production plays a critical role in PH 42. However, whether MT3 affects ROS production in PASMCs during PH has not been reported. Thus, we first examined the effect of MT3 on ROS in HPASMCs infected with MT3-knockdown and MT3-overexpressing lentivirus, respectively. The results showed that although hypoxia induced large amounts of ROS in HPASMCs, neither MT3 knockdown nor overexpression had any additional effect on ROS production, suggesting that the regulation of MT3 on the proliferation of PASMCs may be independent of its antioxidant function **(Figure S4A-S4D)**. It is well known that MT3 is a zinc-binding protein that acts primarily to maintain intracellular Zn^2+^ homeostasis 16, but whether Zn^2+^ is essential for the pro-proliferative role of MT3 on PASMCs remains unknown. To verify the involvement of Zn^2+^ in MT3-associated changes in the proliferative status of PASMCs, a cyto-fluorescence assay was performed to detect intracellular Zn^2+^ levels using Zinpyr-1 (Zn^2+^-targeted fluorescence probe). The results showed that hypoxia increased intracellular Zn^2+^ accumulation, which was likely caused by hypoxia upregulating MT3, as evidenced by the fact that hypoxia-associated intracellular Zn^2+^ accumulation was significantly reversed after we next knocked down MT3 in HPASMCs** (Figure 5A and 5B)**. Flow cytometry after Zinpyr-1 probe loading provided similar results, showing that MT3 knockdown resulted in a significant decrease in hypoxia-induced intracellular Zn^2+^ levels in HPASMCs **(Figure 5C)**. In contrast, MT3 overexpression further increased intracellular Zn^2+^ levels under hypoxic conditions in HPASMCs **(Figure 5D-5F)**. Collectively, these results suggest that MT3 may regulate the proliferative status of PASMCs by altering the homeostasis of intracellular Zn^2+^. To test this hypothesis, MT3 knockdown HPASMCs were treated with exogenous Zn^2+^ (50 μM ZnSO_4_) and cultured under hypoxia for 48 hours. The results of cell counting, CCK-8 and EdU incorporation assays all consistently showed that supplementation of exogenous Zn^2+^ largely restored HPASMC proliferation inhibited by MT3 knockdown under hypoxic conditions, suggesting that Zn^2+^ is essential for the function of MT3 on PASMC proliferation** (Figure 5G-5J)**.

It is well known that metal-regulated transcription factor 1 (MTF1) is a transcriptional regulator whose function is closely related to intracellular Zn^2+^ concentration 43, 44, and Zn^2+^ can mediate MTF1 translocation into the nucleus to exert transcriptional regulation in PASMCs 45. To further investigate whether MTF1 is involved in the function of MT3 on HPASMC proliferation, we knocked down MTF1 in MT3-overexpressing HPASMCs and cultured them under hypoxic conditions for 48 hours. Unexpectedly, MTF1 knockdown significantly counteracted the promotion of HPASMC proliferation by MT3 overexpression, as evidenced by the results of cell counting, CCK-8, and EdU incorporation assays **(Figure 5K-5N)**. Thus, we proposed that Zn^2+^-activated MTF1 was primarily responsible for the pro-proliferative effect of MT3 in PASMCs.

### MT3 Promotes Autophagosome Formation in PASMCs by Upregulating ATG5 Expression

**Figure 6:**
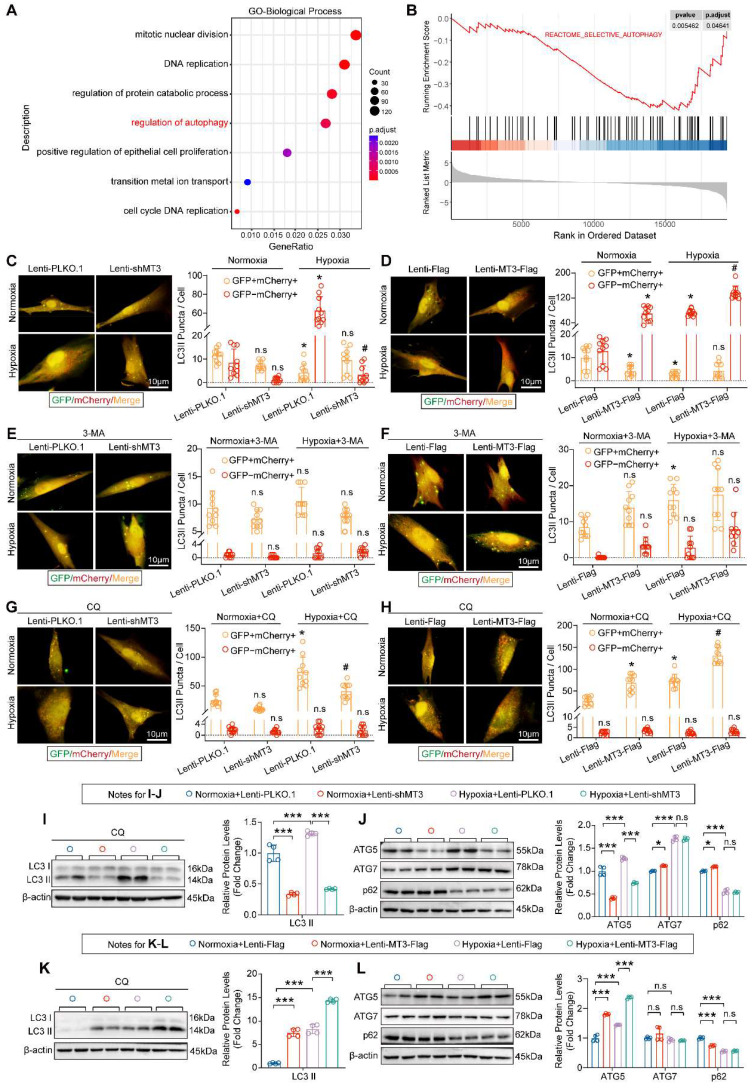
** MT3 promotes autophagosome formation in HPASMCs by upregulating ATG5 expression. A**, Gene Ontology (GO) enrichment analysis showing the biological processes enriched by DEGs between control and MT3 knockdown groups. **B**, Gene set enrichment analysis (GSEA) showing the decrease in autophagic activity. **C**-**H**, Autophagic flux of HPASMCs was detected by infection with GFP-mCherry-LC3-overexpressing lentivirus under the indicated treatments. Yellow dots indicate autophagosomes and red dots indicate autolysosomes. **C** and **D**, Representative images and quantitative analysis showing the autophagic flux activity of MT3 knockdown (or not) or overexpression (or not) followed by normoxic or hypoxic stimulation for 12 hours, respectively (n=10, scale bar, 10 μm). **E**-**H**, Representative images and quantitative analysis were performed under the conditions of **C** and **D** followed by treatment with 3-MA (5 μM) or CQ (10 μM) (n=10, scale bar, 10 μm). For C-H, *P<0.05 versus normoxia+Lenti-PLKO.1 or normoxia+Lenti-Flag or normoxia+Lenti-PLKO.1+3-MA/CQ or normoxia+Lenti-Flag+3-MA/CQ, #*P*<0.05 versus hypoxia+Lenti-PLKO.1 or hypoxia+Lenti-Flag or hypoxia+Lenti-PLKO.1+3-MA/CQ or hypoxia+Lenti-Flag+3-MA/CQ, n.s. indicates no significant difference. **I** and **K**, Western blot and quantitative analysis showed the protein expression levels of LC3 I/II under MT3 knockdown (or not) or MT3 overexpression (or not) and treated with CQ (10 μM) and normoxia/hypoxia for 12 hours (n=4). **J** and **L**, The protein expression levels of ATG5, ATG7, and p62 were detected by Western blot and quantitative analysis in HPASMCs infected with Lenti-shMT3 (or not) or Lenti-MT3-Flag (or not) and normoxia/hypoxia for 12 hours (n=4). *P<0.05, **P<0.01, ***P<0.001, n.s. indicates no significant difference.

Next, to further explore the specific mechanisms and cellular biological processes by which MT3 regulates the proliferation of PASMCs, we subjected the transcriptomics data to GO biological process analysis and GSEA analysis, and the results revealed that the regulation of autophagy pathway was significantly enriched after MT3 knockdown in HPASMCs **(Figure 6A and 6B)**. To further validate these results, we transferred a GFP-mCherry-LC3 plasmid into HPASMCs to monitor autophagic flux. The results showed that the presence of hypoxia led to a significant increase in autolysosomes (shown as red fluorescence by mCherry), which was reduced by MT3 knockdown and further exacerbated by MT3 overexpression, suggesting that MT3 enhanced the hypoxia-induced autophagy in HPASMCs **(Figure 6C and 6D)**. Since autophagy is a dynamic and complex biological process, to clarify the regulatory role of MT3 in autophagy, we first blocked the formation of autophagosomes with 3-methyladenine (3-MA), which was shown to almost reverse the effect of MT3 on hypoxia-induced autophagy **(Figure 6E and 6F)**. Next, chloroquine (CQ) was used to inhibit the fusion and degradation of autolysosomes in cells, resulting in the accumulation of undegraded autophagosomes (shown as a yellow fluorescence of mCherry fusion with GFP). Under these conditions, knockdown of MT3 in HPASMCs alleviated the accumulation of autophagosomes, while overexpression of MT3 further increased the number of autophagosomes **(Figure 6G and 6H)**. Moreover, in the presence of CQ, MT3 knockdown decreased, whereas MT3 overexpression increased, hypoxia-induced upregulation of LC3 II protein levels **(Figure 6I and 6K)**. These results suggest that MT3 was involved in the regulation of hypoxia-induced autophagosome formation in HPASMCs. Since both ATG5 and ATG7 are key regulators involved in the autophagosome formation, we found that ATG5 expression was repressed by MT3 depletion and enhanced by MT3 overexpression under hypoxia, but ATG7 expression was not affected by MT3 **(Figure 6J and 6L)**. In conclusion, we demonstrated that MT3 promoted hypoxia-induced autophagosome formation in PASMCs via upregulation of ATG5 expression.

### Zn^2+^-MTF1-ATG5-regulated Autophagosome Formation is Essential for the Function of MT3 on PASMCs

**Figure 7:**
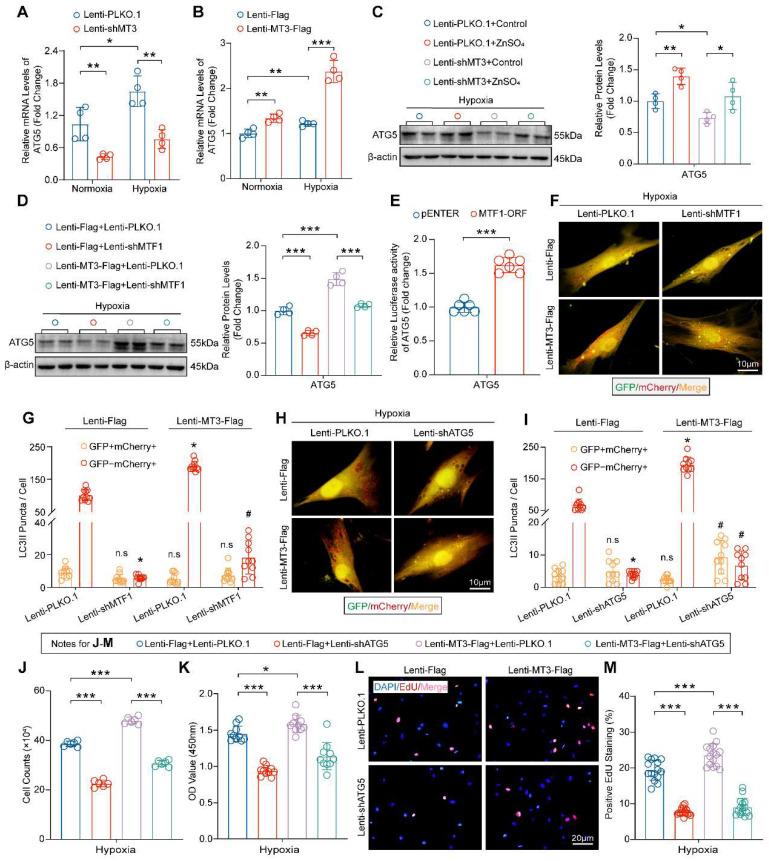
** MT3 promotes HPASMC proliferation via Zn^2+^-MTF1-ATG5 regulation of autophagosome formation. A** and **B**, ATG5 mRNA levels in HPASMCs with MT3 knockdown (or not) or MT3 overexpression (or not) were detected by RT-PCR (n=6). **C**, Western blot and quantitative analysis showing the expression level of ATG5 in control and MT3 knockdown groups of HPASMCs treated with ZnSO_4_ (50 μM) and normoxia/hypoxia for 48 hours (n=4). **D**, Western blot and quantification analysis showed the expression level of ATG5 in MT3-overexpressed (or not) HPASMCs associated with infection of Lenti-PLKO.1 or Lenti-shMTF1 and normoxia/hypoxia treatment for 48 hours (n=4). **E**, Luciferase activity of the pGL3-ATG5 promoter in HEK293T cells overexpressing (or not) MTF1 (n=6). **F**-**I**, Representative images and quantitative analysis of MT3-overexpressing (or not) HPASMCs infected with Lenti-PLKO.1 and Lenti-shMTF1 (**F** and **G**) or Lenti-shATG5 (**H** and **I**) and normoxia/hypoxia for 12 hours showed changes in autophagic flux (n=10, scale bar, 10 μm). **J-M**, HPASMCs first infected with Lenti-Flag or Lenti-MT3-Flag were reinfected with Lenti-PLKO.1 or Lenti-shATG5 and subjected to hypoxia for 48 hours for all groups. The number of HPASMCs was counted after the indicated treatments in **J** (n=6), CCK-8 assay was used to evaluate cell viability in **K** (n=10), and EdU incorporation assay was used to evaluate the proliferative capacity of HPASMCs in **L** and **M** (n=15, scale bar, 20 μm). *P<0.05, **P<0.01, ***P<0.001, For G and I, *P<0.05 versus Lenti-Flag+Lenti-PLKO.1, #*P*<0.05 versus Lenti-MT3-Flag+Lenti-PLKO.1, n.s indicates no significant difference.

Our data have shown that the MT3-Zn^2+^-MTF1 axis promotes PASMC proliferation, and to determine whether ATG5 mediates this effect, we first examined the mRNA level of ATG5 in HPASMCs, and the results suggested that MT3 knockdown decreased and overexpression increased the mRNA level of ATG5** (Figure 7A and 7B)**. More importantly, exogenous Zn^2+^ restored ATG5 downregulation caused by MT3 knockdown and MTF1 deficiency abolished ATG5 upregulation induced by MT3 overexpression in HPASMCs **(Figure 7C and 7D)**. Furthermore, the result of the luciferase reporter assay showed that overexpression of MTF1 (MTF1-ORF) directly enhanced the transcriptional activity of the ATG5 promoter **(Figure 7E)**. Thus, these results suggest that MT3 activates MTF1 by increasing intracellular Zn^2+^ levels, which in turn promotes ATG5 expression.

Next, we are curious about whether MTF1 and ATG5 mediate the role of MT3 on autophagosome formation and PASMC proliferation. The results in Figure 7F-7I showed that knockdown of both MTF1 and ATG5 inhibited autophagosome formation under hypoxic conditions, and further abolished the promotion of autophagosome formation by MT3 overexpression **(Figure 7F-7I)**. Finally, we silenced ATG5 in MT3-overexpressing HPASMCs and the results showed that ATG5 silencing appeared to counteract the hyperproliferation of HPASMCs induced by MT3 overexpression, as demonstrated by cell counting, CCK-8 and EdU incorporation assays after 48 hours of culture under hypoxic conditions **(Figure 7J-7M)**. Taken together, these results suggest that ATG5-regulated autophagosome formation is essential for MT3 to promote PASMC proliferation.

## Discussion

**Figure 8:**
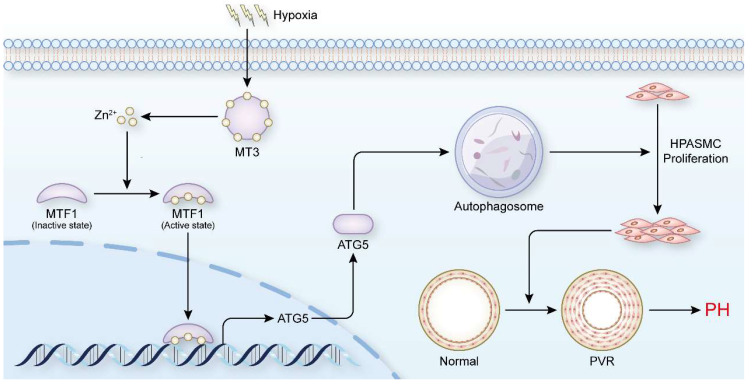
** Schematic summary.** Our findings demonstrate that hypoxia treatment increases the expression of MT3 in HPASMCs, which can release Zn^2+^ and increase the intracellular Zn^2+^ concentration. Zn^2+^ activates MTF1 and mediates MTF1 translocation to the nucleus. MTF1 binds directly to the promoter of the ATG5 gene to facilitate its transcription. ATG5 is a key protein for autophagy initiation and can activate autophagy by mediating autophagosome formation, which in turn promote PASMC proliferation and the development of PH. Therefore, MT3 facilitates PASMC proliferation by promoting Zn^2+^-MTF1-ATG5 axis-mediated autophagosome formation. Thus, targeted inhibition of MT3, Zn^2+^-MTF1 may be promising therapeutic strategies to inhibit pulmonary vascular remodeling and halt the development of PH.

PH is a disease characterized by progressive pulmonary vascular remodeling, and abnormal proliferation of PASMCs is essential for the initiation of pulmonary vascular remodeling 4. Inhibiting the proliferation of PASMCs appears to be a breakthrough in reversing the pathological progression of PH and treating the disease at its core. In the present study, we found that elevated levels of MT3 were detected in tunica media of pulmonary arterioles from PH patients and hypoxia rat/mouse models, as well as in hypoxia-treated human PASMCs. Increased MT3 promoted PASMC proliferation under hypoxic conditions, which was achieved by increasing intracellular Zn^2+^ levels to activate MTF1 transcriptional activity and then upregulating ATG5 expression to facilitate autophagosome formation **(Figure 8)**. Taken together, our results suggest that the MT3-Zn^2+^-MTF1-ATG5 axis is a novel critical pathway involved in PASMC proliferation and PH.

The expression of MT3 in the pulmonary vasculature (e.g., in pulmonary artery endothelial cells and smooth muscle cells) was discovered as early as the late 1990s, although there has been a lack of new studies until a recent report suggesting that MCT may induce upregulation of MT3 in the rat pulmonary microvasculature, but the exact mechanism is still unclear 39, 46-48. In this study, we found that the expression level of MT3 was increased in smooth muscle layer but not endothelial layer of pulmonary arterioles in PH patients and hypoxia-induced rat/mouse PH models. Although comparable expression level of MT3 in endothelial cells was detected between groups, it does not mean that MT3 does not affect the function of endothelial cells involved in the development of PH 38, 49. Moreover, although MT3 has different expression patterns in endothelial cells and PASMCs, there are also interactions between smooth muscle cells and endothelial cells that are jointly contributed to the progression of PH 50. Therefore, further studies are needed to elucidate whether MT3 can regulate endothelial cell function to affect PH. Previous studies have suggested that MT3 is a hypoxia upregulated oncogene, which may be associated with increased hypoxia-inducible factor-1α (HIF-1α) and HIF-2α 22. Similarly in human adipocytes, MT3 was found to be dramatically and rapidly induced by hypoxia and may be transcriptionally regulated by HIF-1 51. However, although HIF-1α is also activated in hypoxia-induced PH models, whether increased MT3 expression during PH is regulated by HIF-1α requires further investigation.

Moreover, we revealed that MT3 promoted PASMC proliferation. This is a very interesting finding because MT3 has long been thought to be a growth inhibitor. For example, Tao et al. found that the promoter of MT3 was hypermethylated and showed a lower expression level of MT3 in patients with acute myeloid leukemia, and MT3 overexpression decreased tumor cell growth and induced apoptosis 21. Tian et al. found similar results in esophageal cancer cells 52. In contrast, Tsui et al. demonstrated that MT3 overexpression promoted the proliferation, invasion and tumorigenesis of bladder cancer cells by downregulating the expression of N-myc downregulated gene 1 (NDRG1), NDRG2, and mammary serine protease inhibitor (MASPIN) gene 22. These studies suggest that there may be differences in the upstream mechanisms that regulate MT3 expression in distinct diseases. The diverse effects of MT3 on disease may be through the regulation of different downstream targets or with different partners. Therefore, potential side effects need to be taken into account when considering MT3 as a drug target.

MT3 is a Zn-binding protein that can bind up to seven Zn^2+^, and is involved in maintaining intracellular Zn^2+^ homeostasis 16, 53. Our results demonstrated that under hypoxia treatment, MT3 knockdown reduced intracellular Zn^2+^ levels and exogenous Zn^2+^ supplementation largely reversed the inhibitory effects of MT3 knockdown on PASMCs. Interestingly, increased free Zn^2+^ is taken up by MTF1 and contributes to the translocation of MTF1 to the nucleus and activation of its transcriptional activity 54, 55. More importantly, accumulating studies suggest that elevated intracellular Zn^2+^ may promote the development of PH. For example, Zhao et al. showed that zinc transporter ZIP12 deficiency decreased intracellular labile Zn^2+^ levels and inhibited proliferation of PASMCs under hypoxia treatment, and ZIP12 knockout attenuated hypoxia-induced PH in rats 56. A recent study showed that elevated intracellular Zn^2+^ led to MTF1 overexpression and activation, which promoted placental growth factor transcription to enhance PASMC proliferation 45. Additionally, Arriaza et al. found that increasing plasma Zn^2+^ concentration under conditions of intermittent low-pressure hypoxia could cause PVR and RVH mediated by MTF1 in Wistar rats 57. Thus, the important role of the Zn^2+^-MTF1 axis in PH is supported by both our results and these published studies. More importantly, we revealed that an endogenous Zn^2+^ regulator, MT3, is required for the proliferation of PASMCs and even for the development of PH.

Interestingly, we further found that the MT3-Zn^2+^-MTF1 axis positively regulated PASMC proliferation by directly promoting ATG5 expression and subsequently autophagosome formation. Although autophagy has been extensively studied in PASMC proliferation and PH, it remains controversial and needs to be further elucidated. It has been reported that chloroquine (CQ), a lysosomotropic agent that inhibits lysosomal degradation during autophagy, increased BMPR2 expression by inhibiting its lysosomal degradation to prevent PASMC proliferation and PH development 11. In addition, ATG5 knockdown inhibited autophagy by directly suppressing the formation of autophagosomes and subsequently inhibited PASMC proliferation in rats 11. Similarly, 3-methyladenine (3-MA), an inhibitor of autophagy initiation, was reported to reverse hypoxia-induced proliferation of PASMCs by regulating autophagy 58. These studies further support our findings that activation of autophagy is a driver of PASMC proliferation and PH development. Conversely, there is also evidence that activation of autophagy may act as a brake on PASMC proliferation and PH. For example, Lee et al. found that LC3B expression was upregulated in lung tissue from both hypoxia-induced mice and PH patients, but the incidence of hypoxia-induced PH was significantly higher in LC3B knockout mice than in control mice 12. In addition, mTOR was able to activate PASMC proliferation by inhibiting autophagosome formation, whereas its inhibitor rapamycin reversed hypoxia-induced PASMC proliferation 13-15. Reasons for this seemingly contradictory result may be: 1) The role of autophagy may be different at different stages of PH progression. Mao et al. argued that autophagy may protect PH by scavenging harmful contents in the early stage of PH, whereas autophagy activation in the mid to late stage of PH may lead to inflammation and metabolic abnormalities, which may contribute to the progression of PH 59; 2) different levels of autophagy may lead to different outcomes; and 3) different steps in disrupting autophagy may have different effects on the development of PH. These findings suggest that targeting autophagy for the treatment of PH needs to be further explored, and that targeting the endogenous regulators of autophagy may be a better alternative strategy than directly targeting autophagy with exogenous drugs for the treatment of PH. Our results suggest that the MT3-Zn^2+^-MTF1 axis may be a potential target.

The overall strengths of our study include: first, we found that the expression levels of MT3 are elevated in hyper-proliferating PASMCs and pulmonary arterioles from patients with PH; second, we demonstrated that MT3 promotes PASMC proliferation by increasing endogenous Zn^2+^ levels and MTF1 transcriptional activity, and then promotes ATG5 expression to facilitate autophagosome formation. Our study is also limited by the fact that the upstream mechanisms regulating MT3 expression during PASMC hyper-proliferation and PH remain unknown; the function of MT3 in PH *in vivo* requires further investigation. In conclusion, our current study demonstrates that MT3 is a previously uncharacterized positive regulator of PASMC proliferation in PH by enhancing Zn^2+^-MTF1-ATG5 axis-regulated autophagosome formation. Our findings not only provide new insights into the mechanism of PASMC proliferation during PH development but are also important for the development of new strategies targeting MT3 and Zn^2+^-MTF1 for the prevention and treatment of PH.

## Supplementary Material

Supplementary figures.
