# Supplementary material for: Metallothionein 3 Potentiates Pulmonary Artery Smooth Muscle Cell Proliferation by Promoting Zinc-MTF1-ATG5 Axis-mediated Autophagosome Formation
Source: Int J Biol Sci. 2024 May 11;20(8):2904–21. doi: 10.7150/ijbs.92992 (PMC11186363; doi:10.7150/ijbs.92992)
Supplement: Supplementary file 1 — Supplementary figures. [file ijbsv20p2904s1.pdf]

Supplementary Material

Metallothionein 3 Potentiates Pulmonary Artery Smooth Muscle Cell Proliferation by Promoting Zinc-MTF1-ATG5 Axis-mediated Autophagosome Formation

Tianxin Xiong[1*], Yi Li[1*], Molin Yang[1], Bo Huo[1], Xian Guo[1], Liyuan Liu[2], Yanxin Huang[2], Xuehai Zhu[1,3], Qinghua Hu[4,5], Xiang Wei[1,3#], Ding-Sheng Jiang[1,3#], Xin Yi[2#]

[1]Division of Cardiovascular Surgery, Tongji Hospital, Tongji Medical College, Huazhong University of Science and Technology, Wuhan, Hubei, China; [2]Department of Cardiology, Renmin Hospital of Wuhan University, Wuhan, China; [3]Key Laboratory of Organ Transplantation, Ministry of Education; NHC Key Laboratory of Organ Transplantation; Key Laboratory of Organ Transplantation, Chinese Academy of Medical Sciences, Wuhan, Hubei, China; [4]Key Laboratory of Pulmonary Diseases of Ministry of Health of China, Wuhan, China; [5]Department of Pathophysiology, School of Basic Medicine, Huazhong University of Science and Technology, Wuhan, China.

***Tianxin Xiong and Yi Li contribute equally to this work**

Correspondence to

Xin Yi, MD

Department of Cardiology, Renmin Hospital of Wuhan University,

Jiefang Road 238, Wuhan, China.

E-mail: yixin2022@whu.edu.cn

Or

Ding-Sheng Jiang, MD

Division of Cardiovascular Surgery

Tongji Hospital, Tongji Medical College

Huazhong University of Science and Technology

1095 Jiefang Ave., Wuhan 430030, China

Tel/Fax: 86-27-6937-8454; E-mail: jds@hust.edu.cn

Or

Xiang Wei, MD

Division of Cardiovascular Surgery

Tongji Hospital, Tongji Medical College

Huazhong University of Science and Technology

1095 Jiefang Ave., Wuhan 430030, China

Tel/Fax: 86-27-6937-8454; E-mail: xiangwei@tjh.tjmu.edu.cn

Supplemental Figures

Supplemental Figure S1

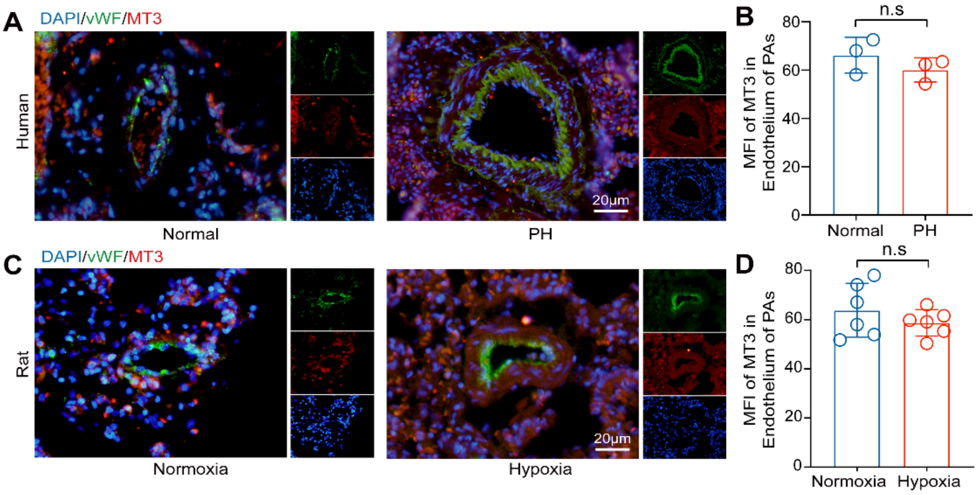

Supplemental Figure S1. MT3 expression level is not altered in the endothelium of pulmonary arteries from PH patients and chronic hypoxia-induced model rat. A, Representative images showing immunofluorescence staining against vWF (green) and MT3 (red) in the pulmonary arteries (PAs) from normal and PH patients (scale bar, 20 μm). **B,** Mean fluorescence intensity (MFI) of MT3 in the endothelium (quantified as the intensity of red fluorescence in the vWF-positive area) of PAs in normal controls and PH patients (n=3). **C,** Representative images showing immunofluorescence staining against vWF and MT3 in PAs from normoxia and 4-week hypoxia-treated rats (scale bar, 20 μm). **D,** Mean fluorescence intensity (MFI) of MT3 in the endothelium of PAs from normoxia- and hypoxia-treated rats (n=6). n.s indicates no significant difference.

Supplemental Figure S2

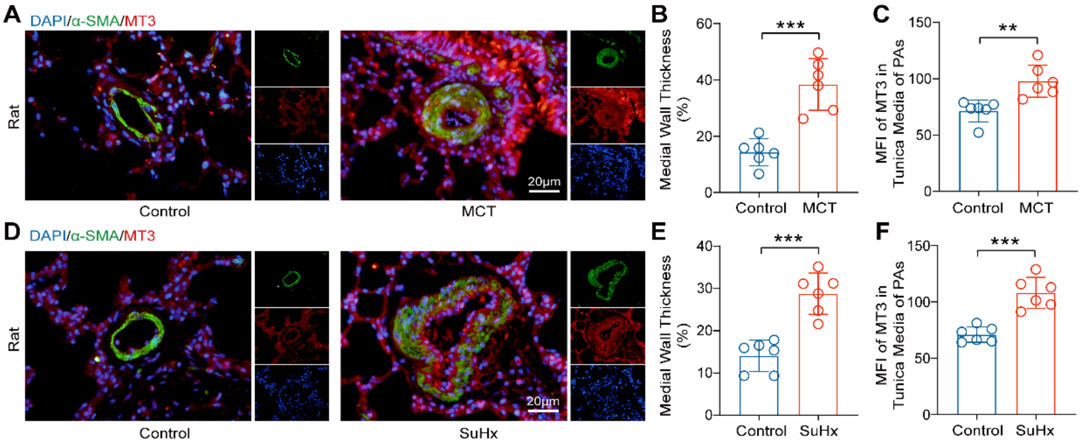

Supplemental Figure S2. MT3 expression level is increased in the tunica media of pulmonary arteries from MCT-induced and SuHx-induced model rats. A, Representative images showing immunofluorescence staining against α-SMA (green) and MT3 (red) from pulmonary arteries (PAs) in control and monocrotaline (MCT)-treated rats (scale bar, 20 μm). **B,** Quantification of tunica medial wall thickness [(outer diameter–inner diameter)/outer diameter] of the PAs in the control and MCT-treated groups, α-SMA marks the tunica media of the pulmonary arteries (n=6). **C,** Mean fluorescence intensity (MFI) of MT3 in the tunica media (quantified as the intensity of red fluorescence in the α-SMA-positive area) of PAs in control and MCT-treated groups (n=6). **D,** Representative images showing immunofluorescence staining against α-SMA and MT3 from PAs in control and Sugen5416 combined with hypoxia (SuHx)-treated rats (scale bar, 20 μm). **E** and **F,** Medial wall thickness and MT3 expression levels in the tunica media of PAs were quantified in control and SuHx-treated rats, and quantitative methods are consistent with **B** and **C** (n=6). **P<0.01, ***P<0.001.

Supplemental Figure S3

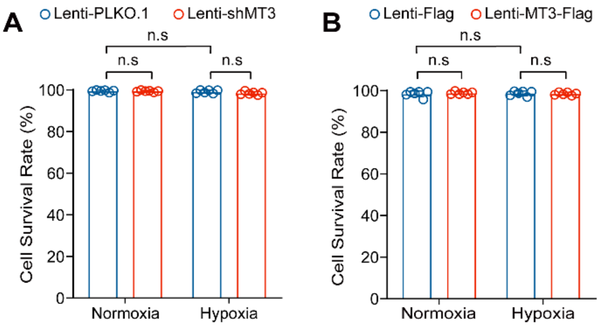

Supplemental Figure S3. Neither overexpression nor knockdown of MT3 affects the cell death of HPASMCs. A and B, Cell survival rate of HPASMCs knockdown (or not) or overexpression (or not) of MT3 subjected to 24 hours of normoxia or hypoxia treatment was assessed by LDH assay (n=6). n.s indicates no significant difference.

Supplemental Figure S4

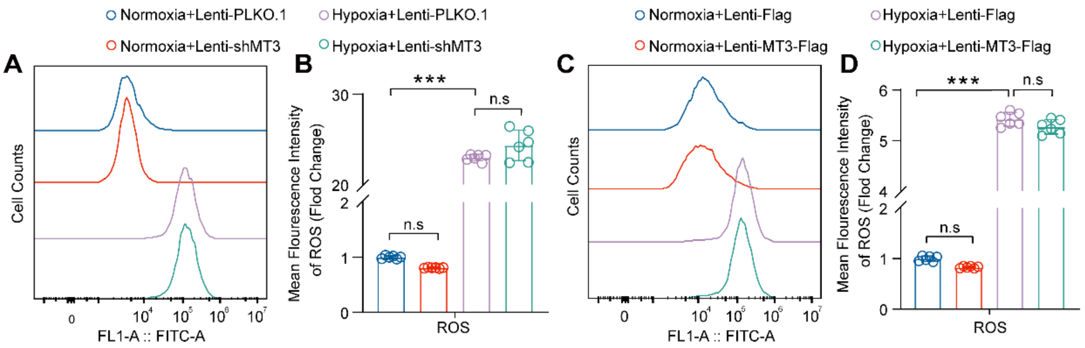

Supplemental Figure S4. The expression level of MT3 does not affects the release of ROS. A and **B**, Representative images and quantitative analysis of mean fluorescence intensity of ROS in HPASMCs of the MT3 knockdown (or not) groups subjected to 48 hours normoxia/hypoxia by flow cytometry (n=6). **C** and **D**, Representative images and quantitative analysis of mean fluorescence intensity of ROS in HPASMCs of the MT3 overexpression (or not) by flow cytometry (n=6). n.s indicates no significant difference, *** P<0.001.
